# Circular of the Committee of Organization of the French Veterinary Congress of 19001Sent to foreign veterinary societies and journals.

**Published:** 1900-03

**Authors:** Ch. Morot


Secretary.

### CIRCULAR OF THE COMMITTEE OF ORGANIZATION OF THE FRENCH VETERINARY CONGRESS OF 1900.[1]

THE Congress of Veterinary Medicine at the Universal Exposition of Paris, September 7 to 11, 1900, inclusive.

A French veterinary congress will be held this year at Paris during the exposition, under the presidency of Senator Dorbot, veterinarian of Langres. It will meet September 7th to 11th, inclusive, at the same time as the international horse-show.

The committee has already received a large number of individual subscriptions in France, besides important subsidies allowed by many French veterinary societies.

The programme embraces the following subjects:

1. Certificates of origin and health.
2. Justification and codification of laws (reasons) for seizure of meats in abattoirs.
3. Organization of a veterinary sanitary service, and the necessity of making it uniform.
4. Production of horses, and the reorganization of studs.
5. The place of veterinarians in agricultural instruction.
6. The management of abattoirs from the sanitary point of view.
7. Knacker's establishments, from the stand-point of the infection of meats, and sanitary police of animals.

Although national (French) the veterinary congress of the exposition will accept with pleasure the membership of those foreign veterinarians who can make their visit to the exposition at the time of its meeting, or of those who wishing to remain in their own country would care to receive the reports and transactions of the congress. All foreign veterinarians who join the congress may take part in the discussions, but only French will have the right of a vote.

The committee trust that foreign veterinarians will consider favorably their appeal and accept their fraternal invitation to take part in the professional work of French veterinarians.

Already a number of foreign veterinarians have sent in their subscription

[1] Sent to foreign veterinary societies and journals.

for membership. Some foreign societies have also subscribed, and decided to send delegates.

The subscription has been fixed at ten francs (two dollars) for foreign and French veterinarians alike, and may be sent to any of the following:

1. M. Larmet, veterinarian of Besancon (Doubs), Secretary-General of the Congress.

2. M. Rossignol, veterinarian of Melun (Seine-et-Moone), member of Committee on Organization.

3. M. le Dr. Moreau, veterinarian of Paris, No. 380 Rue de Vangirard, Paris, Treasurer of the Congress.

4. M. Ch. Morot, veterinarian of Troyes (Aube), Adjunct Secretary of the Congress.

The committee beg that editors of veterinary journals will insert this circular in their next number. The committee also beg that the officers of veterinary societies will call attention of their members to this invitation.

CH. MOROT,
In the name of the Committee.

